# Factors related to turnover intentions and work-related injuries and accidents among professional caregivers: a cross-sectional questionnaire study

**DOI:** 10.1186/s12199-020-00863-8

**Published:** 2020-06-26

**Authors:** Maki Tei-tominaga, Miharu Nakanishi

**Affiliations:** 1 Faculty of Nursing, Setsunan University, 45-1 Nagaotoge-cho, Hirakata City, Osaka, 573-0101 Japan; 2 Research Center for Social Science and Medicine, Tokyo Metropolitan Institute of Medical Science, 2-1-6 Kamikitazawa, Setagaya-ku, Tokyo, 156-8506 Japan

**Keywords:** Professional caregiver, Turnover, Work-related accident and injury, Work environment

## Abstract

**Background:**

The Japanese health and welfare industry has a shortage of professional caregivers, and work-related accidents and injuries among this group are therefore especially critical issues. This study aimed to examine the factors associated with turnover intentions and work-related injuries and accidents among professional caregivers in Japan.

**Methods:**

Self-report questionnaires were distributed to care workers (*N* = 1396) at 26 geriatric-care facilities. The questionnaire addressed basic attributes, work and organizational characteristics, wage adequacy, and intrinsic motivations for work (e.g., “being suited to caring work”). Social-relational aspects of the work environment were assessed via three subscales of the Social Capital and Ethical Climate in the Workplace instrument (i.e., “Social Capital in the Workplace,” “Exclusive Workplace Climate,” and “Ethical Leadership”). Dependent variables were the experience of work-related accidents or injuries in the prior year and organizational and occupational turnover intentions. We used datasets of professional caregivers for analyses.

**Results:**

The response rate was 68% (*N* = 949). Among the 667 professional caregivers, 63% were female. On multivariable logistic regression analysis for work-related accidents and injuries for each sex, those with higher scores for “being suited to caring work” were found to experience significantly fewer work-related accidents and injuries (odds ratio [OR] = 0.78, *p* < 0.01) among female caregivers. Male caregivers who perceived an exclusive workplace climate experienced more work-related accidents and injuries (OR = 1.61, *p* < 0.01). However, experience of work-related accidents and injuries did not show significant relationships with organizational and occupational turnover intentions. Additionally, “being suited to caring work” (OR = 0.73, *p* < 0.01) and ethical leadership (OR = 0.76, *p* < 0.05) were found to be negatively associated with organizational turnover intentions. “Being suited to caring work” (OR = 0.61, *p* < 0.01), inadequacy of wage (OR = 2.22, *p* < 0.05), and marital status (OR = 2.69, *p* < 0.01) were also associated with occupational turnover intentions of professional caregivers.

**Conclusions:**

These findings highlight the need to foster intrinsic motivations for work as well as providing a supportive and ethical work environment to reduce high turnover rates and work-related injuries and accidents among professional caregivers.

## Background

The shortage of professional caregivers is a critical issue in advanced countries [1–3]. Although the number of professional caregivers in Japan has increased 3.3-fold from 2000 to 2017, because of the rapid increase in elderly citizens who need care, the Japanese government has estimated that the shortage of professional caregivers will reach 377,000 in 2025 [2, 3]. In 2018, the job-to-applicant ratio among professional caregivers was 2.5-times higher than the average for all industries [3].

To alleviate the shortage of professional caregivers, measures that address turnover intentions, which are the strongest predictors of turnover behavior [4], are essential. A survey of employers in geriatric-care facilities revealed that the primary reason for a shortage of professional caregivers was the difficulty in hiring, followed by high staff turnover [3, 5]. Additionally, interpersonal relationships in the workplace have been cited by professional caregivers as the main reason for leaving their previous job [5].

Previous studies regarding turnover and turnover intentions among care workers for the elderly in Japan revealed the significance of work and organizational characteristics, including interpersonal relationships. The intention to leave a given organization was associated with poor job role quality, poor skill discretion, high job demands, and poor relationship with supervisors, whereas the intention to leave the occupation was associated with poor skill discretion [6]. Among personal care workers in Australia, perceived supervisor support was identified as a predictor of both intention to leave and intention to stay [7]. Additionally, another study of 2859 care staff members in Japan revealed that those who were in low-turnover facilities were significantly satisfied with their wages, availability of paid days off, and job benefits [8].

Furthermore, to reduce turnover among professional caregivers, measures that address work-related accidents and injuries are also required. The healthcare industry has significantly higher rates of work-related accidents and injuries than do other industries in many countries [9–11]. In Japan, in 2018, most work-related accident, injury, and disease cases (*N* = 5937) occurred within the healthcare and hygiene industry (*N* = 1650) [12]. Workplace injuries are related to turnover intentions of nursing assistants [13], and work-related physical and mental disorders have been cited by professional caregivers as the primary reasons for leaving an organization [3, 5]. A longitudinal study revealed that 30.3% of 1331 workers in nursing homes had had an occupational injury within a 12-month period and the risk ratio of overall job loss within 6 months was 1.31-fold higher among these workers than that among workers who had not experienced injuries [14].

McCaughey et al. [15] identified four antecedents of injury among healthcare employees: individual characteristics, organization of work (such as shift work), job characteristics (such as physical and psychological demands and social-relational aspects of work), and safety programs and training. Another study focusing on the significance of interpersonal relationships in the work environment showed that among 822 hospital nurses, work-related accidents or injuries were significantly associated with working in an exclusive workplace climate (OR = 1.314) [16]. Previous studies of nurses and social workers have also revealed that a positive, ethical workplace climate reduced the intention to leave a job [17] but female nurses react more strongly and favorably toward solid support structures than do male nurses [18]. Similar results may be observed among professional caregivers, as this is also a female-dominated occupation in Japan [19]. Accordingly, when turnover intentions were examined, it is desirable to take into account social-relational aspects of work that may be influenced by sex, as well as the experience of work-related injuries and accidents of the workers.

Thus, this study aimed to examine the factors related to the influence of work-related injuries and accidents considering influence of social-relational aspects of work among professional caregivers, while also examining factors related to turnover intentions, taking into account the influence of work-related injuries and accidents. As few studies have examined these relationships simultaneously, this study provides meaningful insight into occupational safety and health in Japan, where there is a significant shortage of professional caregivers.

## Methods

### Design, settings, and participants

A cross-sectional questionnaire survey was used to collect data from professional caregivers in Japanese geriatric facilities within a 4-month period between August and November 2017. This study used a convenience sampling approach. Twenty-six nursing homes across three prefectures, which were selected on the basis of the ratio of active job openings in long-term care to the number of applicants in 2016 (low, middle, and high ratios of job openings were compared to the national ratio), were invited to participate in this study. The 26 facilities were part of the researchers’ knowledge network pertaining to long-term elderly care. Twelve facilities (46%) were special nursing homes, and 9 facilities (35%) were geriatric intermediate care facilities for the elderly. Self-report questionnaires were distributed to all care workers (*N* = 1396), including professional caregivers, nurses, and care managers, at the nursing homes.

A recent statistical paper presents findings that the sample size for multivariable logistic regression can be determined using information on outcome measures [20]. Additionally, a previous study revealed that events per variable values of 10 or greater indicate no major problems [21]. Referring to these studies, we conducted multivariable logistic regression for each dependent variable after confirming the number of events in each dependent variable.

### Procedures

Each facility that agreed to cooperate with this study was asked to distribute the questionnaires to professional caregivers. The participants were instructed to read the instructions on the face sheet, which included an introductory section consisting of an explanation on the study purpose and voluntary nature of participation and an assurance of anonymity for respondents. The questionnaires were administered over a 4-week period. Completed questionnaires were returned via mail.

### Measures

The dependent variables were experience of a work-related accident or injury and organizational and occupational turnover intentions, which were assessed using three questions (i.e., “Have you experienced a work-related accident or injury within the prior year?,” “Are you planning to quit your current organization by the end of this year?,” and “Are you planning to leave your current occupation by the end of this year?”).

The anonymous self-report questionnaire, which was written in Japanese, also included items addressing individual attributes (i.e., age, sex, marital status, and education) and work and organizational characteristics (i.e., working experience, job qualifications, job position, employment status/type, the region’s job-to-applicant ratio, facility type, average hours of overtime per week, frequency of working on days off per month, duration of experience in current position, and duration of current occupation) that had previously shown significant relationships with the dependent variables [22–24]. Regarding independent variables, one question addressed the perception of adequacy of wage (i.e., “Do you think your wage is sufficient for your work?”) and two questions assessed the intrinsic motivations for work, both of which are important factors for professional caregivers [23, 24]. Additional items queried the presence of a supportive and ethical work environment.

### Intrinsic motivations for work: “being personally suited to caring work”

A previous study of professional caregivers revealed the influence of intrinsic motivations for work (e.g., “I like the elderly” and “I think this is a meaningful job”) on their turnover and retention [23]. Other studies have also revealed that attachment to work is a variable that commonly predicts the intention of nurses to stay in their job [25] and that “being personally suited to nursing work” is the most important factor related to the intention to leave [26, 27]. This study used one subscale (i.e., “Orientation as a Nurse”) of the Nurses’ Job Readiness Scale to assess intrinsic motivations for work. We used two of three items after modifying the words “nursing/nurse” to “caring/caregiver” (e.g., “I utterly hate caring work” and “I don’t have an aptitude for caring work”); each item was evaluated using a Likert scale ranging from 1 (*totally agree*) to 4 (*totally disagree*). Higher total scores indicated a higher intrinsic motivation as a professional caregiver (i.e., the individual felt personally suited to caring work). In this study, Cronbach’s alpha was 0.82.

### Social-relational aspects of work: The Social Capital and Ethical Climate in the Workplace scale

To assess social-relational aspects of the work environment, we used the Social Capital and Ethical Climate in the Workplace (SEW) scale, which has high internal reliability, criterion-related validity, and construct validity [28]. The SEW scale is composed of 20 items developed to evaluate the extent and intensity of associational links or activities, support, reciprocity, and trust among nurses working in Japanese hospitals. It consists of three subscales: “Social Capital in the Workplace” (nine items, e.g., “Overall, nurses are trustworthy”), “Exclusive Workplace Climate” (five items, e.g., “New staff are not readily accepted by the team”), and “Ethical Leadership” (six items, e.g., “Leaders express their understanding of staff nurses’ rights”). Social capital refers to social relationships that facilitate collective action for mutual benefit [29, 30]. Each item was modified to use the words “caring/caregiver” instead of “nursing/nurse” and was evaluated using a Likert scale ranging from 1 (*totally disagree*) to 7 (*totally agree*). We calculated the mean subscale scores among participants. The higher the mean scores of the “Social Capital in the Workplace” and “Ethical Leadership” subscales, the greater the extent to which the participants perceived favorable characteristics of the work environment. In contrast, the higher the mean score of the “Exclusive Workplace Climate” subscale, the greater the extent to which the participants perceived unfavorable work environment characteristics. In this study, Cronbach’s alpha values were 0.93 for Social Capital in the Workplace, 0.95 for Ethical Leadership, and 0.86 for Exclusive Workplace Climate.

### Data analysis methods

First, we calculated the descriptive statistics for individual attributes and work and organizational characteristics, as well as the descriptive statistics and Cronbach’s alpha coefficients of the dependent and independent variables. Next, as a preliminary analysis to identify the primary variables for a multivariable logistic regression analysis, bivariate correlations were calculated between dependent variables (i.e., experience of work-related accidents or injuries within the previous year, plans to quit the organization within the current year, and plans to leave the occupation within the current year) and independent variables (i.e., intrinsic motivation for work: “being suitable for caring work,” perception of amount of wage, three sub-scales of SEW). Additionally, we calculated bivariate correlations between the independent variables. Finally, after confirming the number of events in each dependent variable [21], multivariable logistic regression analysis was performed to examine factors related to the three dependent variables.

Regarding the model for experience of work-related accidents or injuries within the previous year, we conducted a sex-based multivariate logistic regression analysis to examine factors related to the three dependent variables. All independent variables (intrinsic motivation for work: “being suitable for caring work,” perception of amount of wage, and three sub-scales of SEW) were entered into the model after adjusting for basic attributes and employment characteristics selected by the preliminary analysis. Regarding two dependent variables for turnover intentions (i.e., plans to quit the organization within the current year, and plans to leave the occupation within the current year), the abovementioned independent variables and experience of work-related accidents or injuries within the prior year were entered into the equation using a stepwise method (backward selection method) along with variables for basic attributes and employment characteristics selected by the preliminary analysis. ORs and 95% confidence intervals were calculated for each control and independent variable. All statistical analyses were performed using IBM SPSS 25.0 (IBM Japan Ltd., Tokyo, Japan). A *p* value of < 0.05 was regarded as significant.

## Results

Among 1396 care workers, 949 agreed to participate by completing the questionnaire and returning it in a sealed envelope (response rate = 68%). After excluding 44 participants (valid response rate = 94%) who failed to provide the necessary data, we analyzed the datasets of the remaining professional caregivers (*N* = 667).

Of the 667 professional caregivers, 37% were male (mean age = 34.49 years, standard deviation = 8.13) and 63% were female (mean age = 40.43 years, standard deviation = 12.32). The age difference between sexes was significant (*p* < 0.001). Twenty-five percent of male caregivers and 32% of female caregivers answered that they had experienced work-related accidents or injuries within the prior year. Although not shown in the table, we found significant differences between the two sex groups (*p* < 0.05). Regarding organizational and occupational turnover intentions, 8% of male caregivers and 6% of female caregivers answered that they planned to leave their current organization by the end of the year and 7% of male caregivers and 5% of female caregivers answered that they planned to leave their current occupation by the end of the year (Table 1).

**Table 1:** Basic attributes and characteristics of study participants (*N* = 667)^a.^

Variables		Male (*n* = 248)		Female (*n* = 419)		All participants (*n* = 667)	
		*N*	%	*N*	%	*N*	%
1. Marital status	Married	130	52	235	56	365	55
	Single	118	48	184	44	302	45
2. Education	Junior high school graduate	1	0	10	2	11	2
	High school graduate	44	18	158	38	202	30
	Junior college or vocational school equivalency degree	132	53	208	50	340	51
	College graduate	69	28	42	10	111	17
	Others	2	1	1	0	3	0
3. Working experience as an employee	Have	100	40	241	58	341	51
	None	148	60	178	42	326	49
4. Job qualifications	Certified care worker	218	88	348	83	566	85
	Geriatric care manager	19	8	57	14	76	11
	Home helper	11	4	14	3	25	4
5. Job position	Staff	173	70	357	85	530	79
	Unit leader or senior staff member	65	26	46	11	111	17
	Manager in the department	7	3	6	1	13	2
	Others	3	1	10	2	13	2
6. Employment status/type	Regular employee	239	96	312	74	551	83
	Non-regular employee	9	4	107	26	116	17
7. The region’s jobs-to-applicants ratio	Low (1.60 times)	10	4	24	6	34	5
	Middle (2.37–2.79 times)	54	22	158	38	212	32
	High (3.56 times)	170	69	237	57	407	61
8. Facility type	Special nursing homes	107	43	162	39	269	40
	Geriatric intermediate care facilities	137	55	232	55	369	55
	Nursing home with an additional benefit for dementia care	1	0	14	3	15	2
	Others	3	1	11	3	14	2
9. Average hours of overtime per week	≤ 39 h	28	11	128	31	156	23
	40–44 h	121	49	184	44	305	46
	45–49 h	63	25	72	17	135	20
	50–54 h	18	7	21	5	39	6
	≥ 55 h	18	7	14	3	32	5
10. Frequency of working on days off per month	None	182	73	375	89	557	84
	One to two days per month	53	21	38	9	91	14
	Three to four days per month	9	3	5	1	14	2
	More than five days	4	2	1	0	5	1
11. Perception of adequacy of wage	Insufficient at all (less than 80% expected)	135	54	189	45	324	49
	Relatively insufficient (90% expected)	68	27	139	33	207	31
	Sufficient (more than 100%)	45	18	91	22	136	20
12. Experience of work-related accidents or injuries within the prior year	Have	63	25	136	32	199	30
	None	185	75	283	68	468	70
13. Plan to quit current organization by the end of the year	Have	20	8	25	6	45	7
	None	228	92	394	94	622	93
14. Plan to leave current occupation by the end of the year	Have	18	7	23	5	41	6
	None	230	93	396	95	626	94
		Mean	SD	Mean	SD	Mean	SD
15. Age		34.49	8.13	40.43	12.32	38.22	11.31
16. Duration of experience in current position		7.23	5.35	6.74	5.06	6.92	5.17
17. Duration of experience of current occupation		8.72	5.81	8.36	5.51	8.49	5.62

^a^Including missing data

Table 2 shows the results of bivariate correlation for all analyzed variables. All independent variables (intrinsic motivation for work: “being suitable for caring work,” perception of amount of wage, and the three sub-scales of SEW) showed a statistically significant relationship with two dependent variables (i.e., organizational and occupational turnover intentions), while one independent variable (i.e., the experience of work-related accidents and injuries within the prior year) did not show significant relationships with the three sub-scales of SEW. Although not shown in the table, for male caregivers, an exclusive workplace climate, a variable that was a subscale of the SEW scale, showed a statistically significant relationship with the experience of work-related accidents and injuries within the prior year. Additionally, the correlation coefficients between the three sub-scales of the SEW were |0.56|–|0.78|.

**Table 2:** Bivariate correlation for all analyzed variables (*n* = 667)^a^

Variables	(1)^b^	(2)^c^	(3)^d^
(1) Experience of work-related accidents or injuries within the prior year^e^	1.00	< 0.01	0.02
(2) Planning to quit your current organization by the end of the year^e^	< 0.01	1.00	0.85***
(3) Planning to leave your current occupation by the end of the year^e^	0.02	0.85***	1.00
(4) Sex (1, male; 0, female)	0.07^†^	− 0.04	− 0.03
(3) Age	0.13**	− 0.08*	− 0.09*
(4) Marital status (1, married; 0, single)	0.03	0.09*	0.11**
(5) Education (1, less than high school; 0, others)	0.06^†^	− 0.03	− 0.04
(6) Education (1, more than university; 0, others)	− 0.05	− 0.07^†^	− 0.03
(7) Employment status/type (1, regular employees; 0, others)	− 0.02	0.01	0.05
(8) Duration of experience in current position (year)	0.01	< − 0.01	− 0.02
(9) Duration of experience of current occupation (year)	0.04	< 0.01	0.03
(10) Working experience as an employee (1, have; 0, none)	− 0.13***	− 0.02	< 0.01
(11) Job position (1, staff; 0, others)	0.05	0.09*	0.08*
(12) Average hours of overtime per week (1, ≤ 4 h; 0, others)	0.01	− 0.04	− 0.01
(13) Average hours of overtime per week (1, 5–9 h; 0, others)	− 0.01	0.02	0.04
(14) Average hours of overtime per week (1, ≥ 10 h; 0, others)	< − 0.01	0.02	− 0.02
(15) Frequency of working on days off per month (1, ≥ 1 times; 0, others)	< − 0.01	< − 0.01	− 0.01
(16) Intrinsic motivation of work: “being suitable for caring work”^f^	− 0.10**	− 0.12**	− 0.14**
(17) Perception of adequacy of wage (1, insufficient at all; 0, others)	0.11**	0.08*	0.10**
(18) Perception of adequacy of wage (1, relatively insufficient; 0, others)	− 0.03	− 0.09*	− 0.09*
(19) Item mean social capital^f,g^	0.03	− 0.11**	− 0.08*
(20) Item mean ethical leadership^f,g^	0.02	− 0.14***	− 0.12**
(21) Item mean exclusive workplace climate^f,g^	0.05	0.12**	0.12**

^†^*p* < 0.10

*p < 0.05

**p 0.01

***p 0.001

^a^Spearman’s correlation coefficient

^b^To the question “experience of work-related accidents or injuries within the prior year,” 1, have; 0, none

^c^To the question “planning to quit your current organization by the end of the year,” 1, have; 0, none

^d^To the question “planning to leave your current occupation by the end of the year,” 1, have; 0, none

^e^1, have; 0, none

^f^Continuous variables

^g^Sub-scales of the social capital and ethical climate at the workplace

In the results of the multivariable logistic regression model for work-related accidents and injuries according to sex, female caregivers who had higher scores for being suited to caring work (OR = 0.78, *p* < 0.01) experienced fewer work-related accidents and injuries within the prior year, whereas male caregivers who perceived an exclusive workplace climate experienced more accidents and injuries (OR = 1.61, *p* < 0.01) (Table 3). In the results of multivariable logistic regression model for turnover intentions for all professional caregivers, those who had higher scores for being suited to caring work (OR = 0.73, *p* < 0.01) and those who perceived the presence of more ethical leadership (OR = 0.76, *p* < 0.05) were significantly less likely to have plans to leave the current organization by the end of the year (Table 4). Additionally, “being suited to caring work” (OR = 0.61, *p* < 0.01), perception of amount of wage (i.e., insufficient amount) (OR = 2.22, *p* < 0.05), and marital status (OR = 2.69, *p* < 0.01) were found to be associated with occupational turnover intentions of professional caregivers. However, experience of work-related accidents and injuries did not show significant relationships with organizational and occupational turnover intentions (Table 5).

**Table 3:** Multivariable logistic regression model for experience of work-related accidents and injuries within the prior year according to sex^a^

Variables	Male professional caregivers (*n* = 248)^b^			Female professional caregivers (*n* = 419) ^c^		
	Odds ratio	95% confidence interval	*p* value	Odds ratio	95% confidence interval	*p* value
Intrinsic motivation for work: “being suitable for caring work”^d^	0.81	0.81–1.05	0.11	0.78	0.65–0.94	< 0.01
The social capital and ethical climate at the workplace						
Social capital in the workplace	0.76	0.76–1.19	0.24	1.26	0.88–1.79	0.19
Ethical leadership	1.47	1.47–2.21	0.05	1.06	0.81–1.40	0.62
Exclusive workplace climate	1.61	1.61–2.18	< 0.01	1.06	0.85–1.31	0.59

^a^Results of the multivariable logistic regression analysis for experience of work-related accidents or injuries within the prior year. Along with the control variables (age, marital status, working experience, and average hours of overtime per week), independent variables were entered into the equation

^b^Hosmer-Lemeshow goodness of fit *χ*^2^ = 9.554, df = 8, *p* = 0.298

^c^Hosmer-Lemeshow goodness of fit *χ*^2^ = 10.454, df = 8, *p* = 0.235

^d^Continuous variable

**Table 4:** Multivariable logistic regression model for intention to quit current organization by the end of the year (*n* = 667)^a,b^

Variables	Professional caregivers		
	Odds ratio	95% confidence interval	*p* value
Marital status (1, single; 0, married)	0.51	0.25–1.01	0.05
Job position (1, staff; 0, others)	2.82	0.84–9.45	0.09
Perception of amount of wage (1, insufficient at all; 0, others)	1.55	0.80–7.03	0.19
Intrinsic motivation for work: “being suitable for caring work”^c^	0.73	0.57–0.92	< 0.01
Social capital and ethical climate at the workplace			
Ethical leadership ^c^	0.76	0.61–0.95	< 0.05

^a^Results of the multivariable logistic regression analysis for planning to quit current organization by the end of the year. All independent variables (intrinsic motivation for work: “being suitable for caring work,” perception of amount of wage, three sub-scales of SEW, and experience of work-related accidents or injuries within the prior year) were entered into the equation using a stepwise method (backward selection method) along with variables for basic attributes and employment characteristics selected by the preliminary analysis

^b^Hosmer-Lemeshow goodness of fit *χ*^2^ = 2.615, df = 8, *p* = 0.956

^c^Continuous variable

**Table 5:** multivariable logistic regression model for plan to resign from current occupation by the end of the year (*n* = 667)^a,b^

Variables	Professional caregivers		
	Odds ratio	95% confidence interval	*p* value
Marital status (1, married; 0, single,)	2.69	1.27–5.66	< 0.01
Perception of amount of wage (1, insufficient at all; 0, others)	2.22	1.11–4.44	< 0.05
Intrinsic motivation for work: “being suitable for caring work”^c^	0.61	0.48–0.77	< 0.01

^a^Results of the multivariable logistic regression analysis for planning to resign from current occupation by the end of the year. All independent variables (intrinsic motivation for work: “being suitable for caring work,” perception of amount of wage, three sub-scales of SEW, and experience of work-related accidents or injuries within the prior year) were entered into the equation using a stepwise method (backward selection method) along with variables for basic attributes and employment characteristics selected the preliminary analysis

^b^Hosmer-Lemeshow goodness of fit *χ*^2^ = 5.953, df = 8, *p* = 0.545

^c^Continuous variable

## Discussion

The health and welfare industry has significantly higher rates of work-related accidents and injuries (e.g., musculoskeletal disorders) than do other industries [9, 19, 31], and in advanced countries, this sector has a shortage of professional caregivers [1–3]. This study revealed noteworthy findings that are of relevance to these issues and underlined the need to foster intrinsic motivations for work as with providing a supportive and ethical work environment for professional caregivers.

In welfare service facilities for geriatric care in Japan, from 2014 to 2017, “reactionary body movement and/or unreasonable body movement” was found to be the most common cause of work-related accidents and injuries (e.g., back pain) that led to an inability to work for > 4 days [31]. Additionally, male workers aged < 50 years experienced this situation 1.27–1.80 times more frequently than did female workers [31]. However, in our study, female caregivers were significantly more likely to have experienced work-related accidents or injuries in the prior year than were male caregivers. Additionally, male and female professional caregivers differed in their patterns of work-related injuries or accidents in this study.

In our findings for female professional caregivers, “being personally suited to caring work” was associated with a significantly lower risk of work-related accidents and injuries. This suggested that the finding might be attributed to having an attitude to carry out tasks appropriately. In contrast, regarding male professional caregivers, an exclusive workplace climate elevated the risk of work-related accidents or injuries. This result supports that of a previous study, in which an exclusive workplace climate was found to be a risk factor for nurses’ experience of work-related accidents or injuries [16].

Our findings also imply that male professional caregivers might feel exclusion and hesitancy to interact with coworkers because of the lower informal status of male workers in a female-dominated workplace in geriatric care facilities [25], in which > 70% of workers are female caregivers in Japan [19]. Male workers might therefore receive less support from co-workers, which might cause work-related accidents and injuries. A previous review noted that hesitancy to express concerns can be a key contributing factor to communication errors [32]. Another previous study revealed that nurse aides with a higher informal social status had greater access to help from co-workers and reduced exposure to possible injury risk [33].

On the other hand, experiencing work-related accidents and injuries was not a risk factor for organizational and occupational turnover intentions among professional caregivers in our study. These findings are not in agreement with a previous study that found that workplace injuries were related to turnover intentions [13]. Additionally, our finding that intrinsic motivation for work (“being suitable for caring work”) was a consistent protective factor for turnover intentions added a new insight to previous findings.

Kato [23] revealed that intrinsic motivations (e.g., “I like the elderly” and “I think this is a meaningful job”) were significantly associated with intentions to continue working among professional caregivers. Shacklock and Brunetto [25] also demonstrated that attachment to work was a common variable associated with the intention to continue working as nurses across all generations. The researchers also reported that attachment to work is a key for nurses’ retention, and to enable attachment to work to develop, there must be opportunities for nurses to carry out their jobs effectively [25].

Moreover, our finding that higher ethical leadership scores were associated with a reduced likelihood of professional caregiver planning to leave their current organization as a whole adds new insights to the findings of previous reports. A previous study revealed the importance of psychosocial work conditions among employees actually leaving their jobs in Danish eldercare services [34], and our findings also suggest the importance of psychosocial work conditions, especially ethical issues and supervisors’ attitudes towards employees. Ethical issues were reported to be significantly related to nurses and social workers’ intentions to leave their current position [17]. Additionally, support provided by supervisors was found to be an important factor in the turnover intentions of nurses in Australian geriatric care services [7], and the lack of such support was found to increase the risk of turnover among care workers in group homes for elderly individuals with dementia in Japan [35].

The beneficial effects of supervisor support may derive from high-quality leader-member relationships especially in the abovementioned female-dominated workplace. De Hoogh and Den Hartog [36] noted that fair and moral behavior, which they defined as “the concern for morality and fairness” and showed to be associated with trust, is a core component of ethical leadership. While Mueller and Lee (2002) indicated that when high levels of support, trust, and respect exist between supervisors and subordinates, high-quality leader-member exchanges result in an enabling and empowering relationship [37]. Another study revealed that female nurses react more strongly and favorably to robust support structures than do male nurses [18]. Thus, employers and managers in geriatric facilities need to foster a supportive and ethical work environment and especially ethical leadership to intervene regarding turnover intentions among professional caregivers.

Although Kato [23] revealed that relative wages did not show significant associations with intentions to continue working among professional caregivers, wage adequacy, along with marital status, exhibited significant relationships with organizational turnover intentions in this study. Low wage was found to be closely related to a high turnover of professional caregivers in Japan [24]. In addition, regarding worry, anxiety, and dissatisfaction with respect to the work environment among professional caregivers, “low wage” (39.1%) was reported as the second-ranking issue after “lack of manpower” (54.2%) [19]. Our findings suggested that married professional caregivers who need to support family members or who are a dual income family may have occupational turnover intentions, especially in conjunction with inadequate wages. For improvement of worker turnover, further improvements for wages for professional caregivers are required, both within organizations and within presently considered amendments to related laws in Japan [3].

## Limitations

This study has several limitations. First, the participants included only professional caregivers in 26 facilities with 80% of them from special nursing homes and geriatric intermediate care facilities. While special nursing homes and geriatric intermediate facilities are common residential care facilities in Japan, in our sample, the participants were younger (average age: 38.2 years old vs. 43.3 years old), the proportion of females was lower (56% vs. 63%), and the educational level (i.e., junior college or vocational school equivalency degree or higher) was higher (68% vs. 27%) than the professional caregivers in the nationwide survey in Japan [19]. Therefore, the results may be biased and not generalizable to other populations. Second, the cross-sectional nature of the study does not permit one to draw causal inferences; therefore, further research that uses a longitudinal design to examine the effects of supportive and ethical work environments and the aforementioned variables on work-related injuries, accidents, and actual turnover among professional caregivers is required. Third, this study used self-report questionnaires to measure work-related accidents and injuries; hence, the accuracy of our data is unclear. Underreporting of occupational injuries is often noted as an issue by healthcare providers [42]. Finally, while the SEW scale exhibits high internal reliability, further examination is required to confirm its validity.

Notwithstanding these limitations, our findings provide novel insights into occupational health and human resource management in the health and welfare industry in Japan, in which a shortage of professional caregivers and work-related accidents and injuries are critical issues. Further studies are needed to investigate the generalizability of these results.

## Conclusions

This study revealed sex-based difference in factors of work-related injuries or accidents as well as factors of organizational and occupational turnover intentions. The findings highlight the need to foster intrinsic motivations for work as well as providing a supportive and ethical work environment to reduce high turnover rates and work-related injuries and accidents among professional caregivers.

## Data Availability

The data that support the findings of this study cannot be shared publicly because of ethical restrictions imposed by the institutional ethics committee, as the data contain sensitive information on participants and the facilities. However, the data can be made available from the corresponding author for all interested researchers upon requests sent to the author’s office. The initial contact for request should be addressed to the corresponding author’s institution (https://www.setsunan.ac.jp/english/contact/).

## Abbreviations

OR: Odds ratio
SEW: Social Capital and Ethical Climate in the Workplace
